# Effects of APOE genotype on cortical atrophy in early onset Alzheimer’s disease

**DOI:** 10.1002/alz.092060

**Published:** 2025-01-09

**Authors:** Diane Chan, Michael Brickhouse, Alexander Zaitsev, Bonnie Wong, Dustin B. Hammers, Jeffrey L. Dage, Tatiana M. Foroud, Ani Eloyan, Kelly N. Nudelman, Sára Nemes, Maria C. Carrillo, Gil D. Rabinovici, Liana G. Apostolova, Bradford C. Dickerson, Alexandra Touroutoglou, the LEADS Consortium

**Affiliations:** ^1^ Frontotemporal Disorders Unit and Massachusetts Alzheimer’s Disease Research Center, Department of Neurology, Massachusetts General Hospital and Harvard Medical School, Boston, MA USA; ^2^ Massachusetts Institute of Technology, Cambridge, MA USA; ^3^ Massachusetts General Hospital and Harvard Medical School, Boston, MA USA; ^4^ Indiana University School of Medicine, Indianapolis, IN USA; ^5^ Department of Medical and Molecular Genetics, Indiana University School of Medicine, Indianapolis, IN USA; ^6^ Department of Neurology, Indiana University School of Medicine, Indianapolis, IN USA; ^7^ Department of Biostatistics, Brown University, Providence, RI USA; ^8^ Alzheimer's Association, Chicago, IL USA; ^9^ Department of Neurology, Memory and Aging Center, University of California San Francisco, San Francisco, CA USA; ^10^ Memory and Aging Center, Weill Institute for Neurosciences, University of California, San Francisco, San Francisco, CA USA; ^11^ Department of Radiology and Imaging Sciences, Indiana University School of Medicine, Indianapolis, IN USA

## Abstract

**Background:**

APOE‐ɛ4 is a major risk factor for Alzheimer’s disease (AD); its effects have been examined in late‐onset AD (LOAD) but less so in early‐onset AD (EOAD). In LOAD, APOE genotype has strong effects on episodic memory and medial temporal lobe (MTL) atrophy (Wolk & Dickerson, 2010). However, EOAD often presents with more cognitive impairments in executive function, language, and visuospatial abilities than memory. These differences reflect more prominent atrophy in posterior lateral temporal and inferior parietal cortex that mainly constitute the EOAD‐signature of atrophy. Based on the cognitive and neuroanatomical profile of EOAD, we hypothesized that EOAD ɛ4 carriers will have relatively more atrophy in MTL regions subserving episodic memory, whereas non carriers would express more atrophy in cortical regions of the EOAD‐signature involved in executive function, language, and visuospatial abilities including inferior parietal and posterior temporal regions. We also expected worse performance on episodic memory tests in ɛ4 carriers with EOAD.

**Methods:**

We examined the effects of APOE genotype on cortical atrophy and episodic memory of 144 ɛ4 carriers and 117 ɛ4 non‐carriers with EOAD from the Longitudinal Early‐Onset Alzheimer’s Disease Study (LEADS). Between‐group comparisons using independent T‐tests were made for morphometric measures of cortical atrophy in MTL and hippocampus localized in LOAD as well as in cortical regions within our newly developed EOAD‐Signature tool (Touroutoglou et al., 2023). ANCOVA with Bonferonni’s correction was used to evaluate for effects of age on significant differences between groups.

**Results:**

As predicted, ɛ4 carriers with EOAD had more atrophy in the MTL and bilateral hippocampi, whereas non‐carriers had more atrophy in regions of the EOAD‐signature including bilateral caudal temporal, parietal lobule, middle frontal gyrus, mid temporal, posterior cingulate cortex, precuneus, superior frontal gyrus, superior parietal lobule. Post hoc vertex wise cortical maps further confirmed the specificity of the results. In addition, ɛ4 carriers had worse performance on episodic memory testing (AVLT delayed recall). These results were not explained by a difference in age between the groups.

**Conclusions:**

These results are consistent with prior work (Nemes et al. 2023) and support the hypothesis that the ɛ4 genotype modulates distinct neuroanatomic phenotypes of AD in EOAD patients.

